# Exploring the Benefits of Dog-Assisted Therapy for the Treatment of Complex Trauma in Children: A Systematic Review

**DOI:** 10.3390/children11081017

**Published:** 2024-08-20

**Authors:** Rebekah L. Chapman, Caitlin Baselmans, Tiffani J. Howell, Carol Ronken, David Butler

**Affiliations:** 1 Bravehearts, Arundel, QLD 4214, Australia; chapman.rebekah@police.qld.gov.au (R.L.C.); research@bravehearts.org.au (C.R.); 2 Queensland Police Service, Brisbane, QLD 4000, Australia; 3 Faculty of Psychology, Counselling, and Psychotherapy, The Cairnmillar Institute, Hawthorn East, VIC 3123, Australia; caitlin@psychspan.com.au (C.B.); david.butler@cairnmillar.edu.au (D.B.); 4 School of Psychology and Public Health, La Trobe University, Bendigo, VIC 3552, Australia

**Keywords:** animal-assisted therapy, human–animal relationships, post-traumatic stress disorder, child mental health

## Abstract

Background: The manifestation of complex trauma results from exposure to severe and repetitive stressors occurring within the caregiver system. Frequently associated with child maltreatment, complex trauma can lead to impairments in multiple domains, including attachment, affect and behavioural regulation, and cognition. Treatments, including Trauma-Focused Cognitive Behavioural Therapy, have been shown to be efficacious, but high attrition rates point to the need for complementary methods that boost client retention and treatment efficacy. This systematic review examines whether dog-assisted therapy has beneficial impacts on the treatment of complex trauma and whether it can decrease treatment attrition among children presenting with complex trauma exposure. Methods: We followed PRISMA guidelines to locate relevant research reports. Seven published research reports matched the inclusion criteria. Results: Dog-assisted therapy may increase the efficacy of standard interventions for complex trauma, although only one study formally assessed treatment retention. Across most studies, there is an overall lack of detailed information on the nature of therapeutic activities involving the dog and how these activities may complement or enhance therapy as usual. Studies were of varying methodological quality, impacting the reliability of findings. Conclusions: Future studies should aim to better describe and justify dog-assisted therapy techniques and evaluate these in comparison with standard evidence-based approaches to the treatment of childhood complex trauma.

## 1. Introduction

The term ‘complex trauma’ refers to a dual problem of exposure and manifestation [1]. Complex trauma exposure is the experience of severe and repetitive interpersonal stressors occurring within the caregiver system, beginning in childhood or adolescence [2]. It is a widespread problem: in the United States, 62% of adults surveyed report exposure to at least one adverse childhood experience, most commonly emotional abuse (34%) [3]. Similarly, 62% of the Australian population report having experienced at least one type of maltreatment in childhood, with exposure to domestic violence most reported (40%), followed by physical abuse (32%), emotional abuse (31%), sexual abuse (29%), and neglect (9%) [4]. Multi-type maltreatment is also common, with 39% of Australians having experienced more than one form of childhood abuse [4]. 

The manifestation of complex trauma refers to the presenting symptomology that results from the ongoing impact of these interpersonal stressors on psychological, social, and emotional development [5]. The impact of complex trauma exposure, which frequently occurs during critical developmental periods, may result in the manifestation of impairment across multiple domains, including attachment, physiology (e.g., impaired brain development), affect regulation, dissociation, behavioural regulation, cognition, and self-concept [6]. In addition to increased efforts at preventing maltreatment and identifying abuse in children who are showing complex trauma symptomology, it is imperative that research continues to investigate effective treatment options for children who have been exposed to prolonged, developmentally adverse traumatic events.

There is a wide range of therapeutic treatments that have been developed for children who have experienced complex trauma, and they vary in their level of research-based support. Approaches include Eye Movement Desensitisation and Reprocessing (e.g., [7,8,9]), Parent–Child Interaction Therapy (e.g., [10]), Child–Parent Psychotherapy (e.g., [11]), and Trauma-Focused Integrative Play Therapy [12]. To date, Trauma-Focused Cognitive Behavioural Therapy (TF-CBT) is the approach with the strongest empirical support for the treatment of symptoms associated with complex trauma, with youth receiving TF-CBT showing, for example, reduced post-traumatic stress and depression and improved general mental health symptoms [13,14,15,16,17]. One major concern with current best practise approaches, however, is client attrition. A meta-analysis of TF-CBT with children found that the overall rate of attrition was 34%, with rates across studies ranging from 8% to 69% [18]. Among possible trauma-related factors, clients’ avoidance of distressing trauma-related content, as well as decreased trust in relationships resulting from interpersonal harms, have been posited as key risk factors for therapy attrition [18,19]. There is a need for evidence-based treatments with clinical utility for complex trauma that minimise client avoidance, contribute to the therapeutic alliance, and maximise therapy engagement and completion. One increasingly popular approach is animal-assisted therapy (AAT), and AAT is particularly promising as it can be integrated with other forms of evidence-based treatments, including TF-CBT.

AAT refers to a goal-directed, structured therapeutic treatment whereby an animal is an integral part of the treatment process [20]. In AAT, the animal augments the existing therapy session and is integrated into the treatment process, while the direct focus remains on the objectives of treatment and the healing process of the client [21]. Although theoretical variation does exist (which may not be mutually exclusive), it is commonly proposed that animals facilitate social relationships and empathy, which can, in turn, facilitate relationships and empathy with humans [22,23]. Increasingly, research is focusing on the efficacy of AAT for survivors of trauma. AAT practitioners have primarily employed dogs as part of the treatment process. Consistent with the proposal that AAT may facilitate social relationships and empathy, research has shown that interactions with dogs decrease the human stress response through reductions in cortisol, blood pressure, and heart rate [24]. Moreover, constructive interactions with therapy dogs, who tend to show unconditional positive regard, can influence a trauma survivor’s perspective of themselves as someone who is worthy of affection and trust, and connections with animals may be easier to form for people who struggle with human interactions [25]. It is possible that these benefits may result in reduced attrition from the therapeutic program. Indeed, a meta-analysis of attrition in AAT found overall attrition rates of just 11.2% among the 57 studies included in the meta-analysis, which reported dropout rates [26]. This meta-analysis included all species working in AAT and adult and child populations with varying mental health concerns, so it is possible, but not yet clear, whether this would apply specifically to dog-assisted therapy for child survivors of complex trauma. 

Prior research has looked at the effects of AAT on a diverse range of mental health issues. A 2015 systematic review investigating empirical literature on AAT for trauma, including single-incident as well as complex trauma in both children and adults, reported several benefits, including decreased depression, PTSD symptoms, and anxiety [27]. However, the 10 included studies included both dogs and horses and generally had low methodological rigour (e.g., over-reliance on self-report, with few physiological or behavioural measures included to corroborate the findings [27]). Further, in 2017, Hoagwood et al. [28] conducted a systematic review of AAT for a broad range of mental health issues in children and adolescents, finding that structured therapeutic interventions incorporating either horses or dogs may lead to improved functioning, particularly for specific conditions, including childhood trauma. They indicated that future research should aim to understand the mechanisms of change, which are currently unclear. A recent systematic review from 2022 investigated the impact of dog-assisted therapy on children with behavioural and developmental disorders, but not specifically focused on trauma survivors [29]. It found that, while the results are promising, there are still few high-quality research studies conducted in this area, with information about the dog’s characteristics often lacking, and only a few studies mentioning the use of a protocol or manual for the treatment program [29]. A fourth systematic review of dog-assisted psychotherapy for the mental health treatment of adolescents with issues ranging from complex trauma exposure to diagnoses of anxiety, schizophrenia, and mood disorders found positive impacts on primary diagnoses and symptomology, as well as increased engagement and socialisation behaviours, but again, found a generally low level of methodological rigour [30]. There was considerable variation in the nature of the therapy programs, and which were often poorly described [30]. 

In summary, research involving AAT indicates that it can be an effective means to reduce various mental health issues, including complex trauma. To date, however, there have been no published reviews that have specifically examined the impact of dog-assisted therapy on the treatment of complex trauma in children, with prior reviews including various animal species and both single-incident and complex trauma across broad age ranges. All the information in these prior reviews is instructive, but it is not currently possible to separate out the specific impacts of dog-assisted therapy on child survivors of complex trauma, per se. This means that the potential benefits of dog-assisted therapy for this population, as well as any limitations of existing research in this area which could be used to improve the evidence base in the future, are not entirely clear. Given the increasing use of dog-assisted therapy (with dogs among the most popular species used for AAT) and its potential benefits, and the unique relational and avoidance-related challenges posed by complex trauma, it is imperative to determine whether dog-assisted therapy will be of actual benefit to those suffering complex trauma. As dog-assisted therapy may complement existing evidence-based treatments, it is possible that the inclusion of dogs in therapy may enhance treatment benefits. An additional benefit may be increased therapy completion rates due to the physiological (i.e., reduced stress) and social effects it may facilitate.

A common and ongoing criticism within the field of AAT is the lack of standardisation among AAT programs, as well as the limited description in the research literature of programs and their key components, including the specific role of the animal within these program components [22,31]. This lack of specification of AAT programs limits both the ability to effectively review outcomes across studies and prohibits the broader uptake of effective program elements into therapeutic practise. To best understand the ways in which dog-assisted therapy may complement treatment and impact on complex trauma symptomology, it is imperative that the nature and specific components of effective programs are comprehensively described in the research. If this is currently not available in existing research, a systematic review would help clarify this lack of knowledge and highlight the need for future research to address this limitation, thus strengthening the evidence base.

The aims of this systematic review are therefore three-fold: assess (i) research quality, including consistency in and comprehensiveness of descriptions of dog-assisted therapy program theory and related components; (ii) whether dog-assisted therapy is an effective treatment for children presenting with complex trauma exposure, including whether it has any beneficial impact over and above standard therapeutic treatments; and (iii) whether dog-assisted therapy can decrease attrition and increase therapy completion rates among children. 

## 2. Materials and Methods

### 2.1. Search Strategy

A comprehensive database search was conducted to identify dog-assisted therapeutic treatment of complex trauma in children, in accordance with guidelines from the Preferred Reporting Items for Systematic Reviews and Meta-Analyses (PRISMA; [32]) and the Cochrane Handbook for Systematic Reviews [33]. The aim was to identify all empirical studies that have explored outcomes from dog-assisted therapy for children who have experienced one or many forms of complex trauma exposure, including physical and sexual abuse, neglect, and family violence. Studies were eligible for inclusion if they met the following criteria:

- The dog is included, with or without other animals, as part of a treatment-based, goal-directed intervention that is focused on treating the symptoms of complex trauma.
- Participants are children aged under 18 years who have experienced complex trauma exposure.
- Empirical evidence is provided on quantitative or qualitative outcomes relating to the child’s trauma symptomology.
- Available in English.

A systematic electronic search for eligible studies was conducted using Scopus, Web of Science, and Google Scholar, all on 29 January 2022. Electronic searches of the grey literature database World Cat were also conducted. These search engines were selected based on the combined depth and breadth of subject fields that they include. 

**Figure 1:**
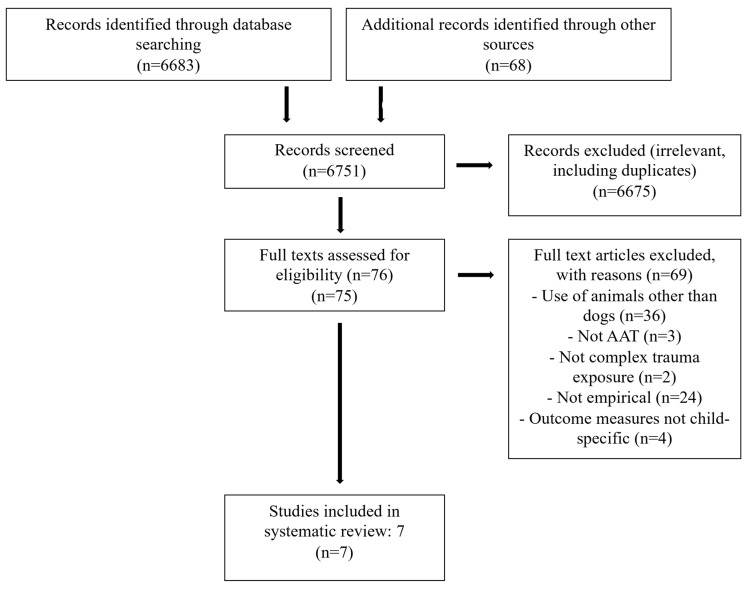
Flow diagram of study selection process.

Commonly used search terms from the AAT and complex trauma literature were individually inserted into the search field ‘keyword, title and abstract’, as detailed in Table 1. Search terms for all databases included at least one term for AAT or dog-assisted therapy and at least one term for trauma or abuse. No restrictions were made regarding the date of publication to ensure a thorough search of all available literature. For each set of search terms entered, the titles of sources were reviewed for relevance by C.B. (i.e., an explicit mention of AAT or dog-assisted therapy, along with complex trauma, interpersonal trauma, or trauma-related symptomology). Abstracts were then examined by the first author, and if not excluded at that stage, full texts were read in depth by C.B. to ensure that the eligibility criteria were met. For all articles meeting the inclusion criteria, reference lists were screened for possible additions. A flow diagram of the study selection process is shown in Figure 1.

### 2.2. Data Extraction and Evaluation

For each identified study, author CB extracted data, which included the following: study aim(s); participant, dog, and handler characteristics; program description, including length, strategies used, and methods by which a dog is integrated into the approach; study design, including identification of a comparison condition if relevant; assessment measures utilised; and outcomes and study results relating to child trauma symptomology, as well as to client retention and therapy completion. Data extraction focused on results specifically showing a change in trauma symptomology from pre- to post-intervention and comparisons in change, where relevant, across intervention and comparison conditions. If effect sizes were not reported in the original studies, these were determined using the online calculator Psychometrica [34]. To allow for a standardised comparison across studies, all effect sizes are reported using Cohen’s d, where 0.2 is a small effect, 0.5 is a medium effect, and 0.8 is a large effect size [34]. A meta-analysis was not possible due to the heterogeneity of the measures and methods. 

Studies were assessed for methodological quality using the National Institute of Health (NIH) guidelines for controlled intervention studies and pre-post studies and the Critical Appraisal Skills Program (CASP) for qualitative research. Each study was rated ‘good’, ‘fair’, or ‘poor’ for confidence in methodological quality. 

## 3. Results

From a total of 6683 screened sources, seven studies, all published between the years 2006 to 2021, were included for synthesis, comprising six quantitative studies and one qualitative observational case study (see Table 2 for details). Three studies were conducted in the USA, two were conducted in Spain, and one each in Israel and Australia. 

The total number of children across the studies was 307, including 220 females (73%). Participants ranged in age from 5 to 17 years. Ethnicity was reported for 206 of the participating children, with 44% being Caucasian, 32% Hispanic, 16% African American, 3% Indigenous Australian, 2% other (including Native American), and 2% multiple ethnicities. All of the participating children were reported to have experienced some form of maltreatment or traumatic childhood experiences, including sexual and physical abuse and exposure to domestic violence, except nine participants in Hamama et al.’s [35] research, who had not experienced child maltreatment but were included as a study comparison group.

**Table 2:** Summary of results for studies investigating dog-assisted therapy in the treatment of child and adolescent trauma.

First Author (Year)	Research Question	Participant(s)	Dog(s) and Handler(s)	Study Design	Program Description and Comparison Group	Measures and Data Collection Time Frames	Results	Quality
Allen et al., 2021 [36]	Does integration of AAT into TF-CBT for abused youth (a) impact retention, (b) present challenges to implementation, (c) demonstrate efficacy?	N = 33 (66% female); 6–17 years. 64% Caucasian, 21% African American, 15% multiple ethnicities. At least one incident of physical, sexual abuse or witnessing inter-partner violence	N = 5 labrador retrievers trained as assistance dogs; handlers observed sessions from behind one-way mirror.	RCT: 2 (time) × 2 (group) design	Individual TF-CBT including assistance dog (TF-CBT + AAT): 12 × 90 min sessions with integration of assistance dog (child-led; clinician also offered interaction with dog as a coping skill during sessions). n = 17. Comparison group: Individual TF-CBT: 12 × 90-min sessions. n = 16.	UCLA PTSD reaction index for DSM-5: caregiver and self-report. Measures post-traumatic stress symptoms. Strengths and difficulties questionnaire (SDQ): caregiver report. Measures youth emotional and conduct problems. Moods and feelings questionnaire (MFQ): self-report. Measures depressive symptoms. Screen for child anxiety-related disorders (SCARED): self-report. Measures anxiety-related concerns. Retention: % dropout. Measures 1–4 administered at Times 1 (baseline), 2 (session 5), 3 (session 9), and 4 (post-intervention). Measure 5 assessed at Time 4.	TF-CBT + AAT (Measures 1–4: baseline (T1) vs. post-intervention (T4)) PTSD: - Caregiver report: T4 < T1 (d = 1.21) - Self-report: T4 = T1 (d = 0.07) 2. SDQ: - Emotional problems: T4 < T1 (d = 1.33) - Conduct problems: T4 < T1 (d = 0.35) 3. MFQ: T4 = T1 (d = 0.01) 4. SCARED: T4 = T1 (d = 0.26) 5. Retention: 6.7% dropout TF-CBT (Measures 1–4: T1 vs. T4): PTSD: - Caregiver report: T4 < T1 (d = 1.67) - Self-report: T4 < T1 (d = 0.86) 2. SDQ: - Emotional problems: T4 < T1 (d = 0.89) - Conduct problems: T4 < T1 (d = 0.35) 3. MFQ: T4 = T1 (d = 0.58). 4. SCARED: T4 < T1 (d = 0.76). 5. Retention: 21.4% dropout. TF-CBT + AAT vs. TF-CBT (Measures 1–4: difference between group change, T1 vs. T4). PTSD: - Caregiver report: TF-CBT + AAT = TF-CBT (d = 0.21) - Youth report: TF-CBT < TF-CBT + AAT (i.e., showed greater decrease) (d = 0.43). 2. SDQ: - Emotional problems: TF-CBT + AAT = TF-CBT (d = 0.14) - Conduct problems: TF-CBT + AAT = TF-CBT (d = 0.03) 3. MFQ: TF-CBT + AAT = TF-CBT (d = 0.12). 4. SCARED: TF-CBT + AAT = TF-CBT (d = 0.07) 5. Retention: TF-CBT + AAT = TF-CBT (d = 0.40).	Good
Balluerka et al., 2015 [37]	Does animal-assisted psychotherapy impact the psychosocial adaptation of adolescents in residential care who have suffered traumatic childhood experiences?	N = 63 (38% female); 12–17 years. Ethnicity not reported (although, 68% of participants were from Basque County, 32% were foreign unaccompanied minors from northern Africa). In residential care; had suffered traumatic childhood experiences (not defined).	N = 1 dog trained as therapy dog. No information on breed or handler identified.	Non-randomised control; 2 (time) × 2 (group) design	Individual psychotherapy (IP) + animal-assisted psychotherapy (AAP): 12-week program (2 days/week, farm-based, separate from and concurrent to individual psychotherapy); 23 group and 11 individual sessions. Five sessions involved integration of therapy dog (including child-led interaction); separate sessions involved horses (number of sessions with horses not specified). n = 43. Comparison group: Individual psychotherapy (IP): dose not specified. n = 24.	1. Behavior Assessment System for Children (BASC, Spanish version): residential carer, teacher, and self-report. Measures indicators of psychosocial adaptation across 5 composite subscales: (a) Clinical maladjustment (including atypicality, locus of control, somatization, anxiety): self-report (b) Behavioural symptoms (including aggression, hyperactivity, attention problems, atypicality, depression, anxiety): teacher and residential carer report (c) Personal adjustment (including interpersonal relations, relations with parents, self-reliance, self-esteem): self-report (d) Adaptive skills (including social skills and leadership): teacher and residential carer report (e) School maladjustment (including attention problems, learning problems, attitudes toward school and teachers, sensation-seeking): teacher and self-report Measure 1 administered at Time 1 (baseline) and Time 2 (Post-intervention).	AAP + IP (Measures 1a–1e: baseline (T1) vs. post-intervention (T2)): 1a. Clinical maladjustment: T2 = T1 (d = 0.44) 1b. Behavioural symptoms: - Teacher report: T2 = T1 (d = 0.28) - Residential carer report: T2 = T1 (d = 0.48) 1c. Personal adjustment: T2 = T1 (d = 0.39) 1d. Adaptive skills: - Teacher report: T2 > T1 (d = 0.87) - Residential carer report: T2 > T1 (d = 0.59) 1e. School maladjustment: - Teacher report: T2 = T1 (d = 0.61). - Self-report: T2 = T1 (d = 0.34). Retention—not reported AAP vs. IP (Measures 1a–1e: AAP change score vs. IP change score). 1a. Clinical maladjustment: AAP = IP (d = 0.04). 1b. Behavioural symptoms: - Teacher report: AAP = IP (d = 0.19) (Note for hyperactivity subscale, AAP < IP: greater reduction, d = 0.98). - Residential carer report: AAP = IP (d = 0.04) 1c. Personal adjustment: AAP = IP (d = 0.09) 1d. Adaptive skills: - Teacher report: AAP > IP (greater positive change, d = 0.91) - Residential carer report: AAP = IP (d = 0.38) 1e. School maladjustment: - Teacher report: AAP = IP (d = 0.54). - Self-report: AAP = IP (d = 0.07). Retention—not reported	Fair
Dietz et al., 2012 [38]	Is standard group therapy, dog-assisted therapy, or dog-assisted therapy with therapeutic stories more effective in the treatment of trauma symptoms for survivors of child sexual abuse?	N = 153 (93% female); 7–17 years. 37% Caucasian, 43% Hispanic, 17% African American, 3% other (including Native American). Confirmed sexual abuse.	No information on dog(s); external therapy dog organisation with handlers attended sessions for initial 10–15 min.	Non-randomised control; 2 (time) × 3 (group) design.	Group therapy (12 sessions) with two intervention types: - Dogs + no stories (DNS): Visits by therapy dogs and their handlers once per month for an average of four visits. Dogs and handlers available in waiting room for 30 min prior to group sessions; joined group for initial 10–15 min. n = 60. - Dogs + stories (DS): As per dogs + no stories, but with addition of stories written by clinical director and incorporated into group program. n = 61. Comparison group: Group therapy (12 sessions). n = 32.	1. Trauma Symptom Checklist for Children (TSCC). Measures trauma symptomology across six clinical subscales (all self-report): (a) Anxiety (b) Depression (c) Anger (d) PTSD (e) Dissociation (f) Sexual concerns. Measure 1 administered at Time 1 (baseline) and Time 2 (Post-intervention).	DS (Measures 1a–1f: baseline (T1) vs. post-intervention (T2)). 1a. Anxiety: T2 < T1 (d = 0.78) 1b. Depression: T2 < T1 (d = 0.87) 1c. Anger: T2 < T1 (d = 0.66) 1d. PTSD: T2 < T1 (d = 0.85) 1e. Dissociation: T2 < T1 (d = 0.83) 1f. Sexual concerns: T2 = T1 (d = 0.59). DNS (Measures 1a–1f: baseline (T1) vs. post-intervention (T2)). 1a. Anxiety: T2 < T1 (d = 0.21) 1b. Depression: T2 < T1 (d = 0.46) 1c. Anger: T2 = T1 (d = 0.16) 1d. PTSD: T2 < T1 (d = 0.35) 1e. Dissociation: T2 = T1 (d = 0.21) 1f. Sexual concerns: T2 = T1 (Cohen’s d = 0.08). Retention—not reported Group therapy (Measures 1a–1f: baseline (T1) vs. post-intervention (T2)). 1a. Anxiety: T2 = T1 (d = 0.09). 1b. Depression: T2 = T1 (d = 0.22) 1c. Anger: T2 = T1 (d = 0.01) 1d. PTSD: T2 = T1 (d = 0.28) 1e. Dissociation: T2 = T1 (d = 0.02) 1f. Sexual concerns: T2 = T1 (d = 0.12). Retention—not reported DS c.f. DNS vs. Group therapy (Measures 1a–1f: Dogs with stories change score vs. Dogs no stories change score vs. Group therapy change score). 1a. Anxiety: DS < (i.e., decreased to greater extent) DNS (d = 0.62). DNS < Group therapy (d = 0.16) 1b. Depression: DS < DNS (d = 0.50). DNS < Group therapy (d = 0.14). 1c. Anger: DS < DNS (d = 0.54). DNS < Group therapy (d = 0.19). 1d. PTSD: DS < DNS (d = 0.46). DNS < Group therapy (d = 0.12). 1e. Dissociation: DS < DNS (d = 0.58). DNS < Group therapy (d = 0.21). 1f. Sexual concerns: DS < DNS (d = 0.43). DNS = Group therapy (d = 0.05). Retention—not reported	Fair
Eggiman 2006 [39]	Will the use of a therapy dog, integrated with CBT, improve PTSD symptomology in a female client with a history of physical and sexual abuse?	N = 1 (female); 10 years. Ethnicity not reported. History of physical and sexual abuse.	N = 1 poodle trained as therapy dog. Therapist is handler.	Single case study	Individual CBT including therapy dog. Initial session involved: explanation of rules, getting to know dog, petting dog and staying relaxed, talking to dog, telling a therapeutic story about dog’s behaviour, and telling him goodbye. No further sessions described; number of sessions not specified.	Behavioural observations only: therapist and carer report. Behaviours: - In session: engagement, attention and focus, relaxation, respect for rules and boundaries, aggression - At home: bedtime routine behaviour, rule-following, aggression, behaviour with animals. Informal observations at Time 1 (Baseline) and Time 2 (during or after therapy).	Observations (T1 vs. T2) In session: - Session engagement: T2 > T1 - Disclosure of abuse memories: T2 > T1 - Attention and focus: T2 > T1 - Rule-following and respect for boundaries: T2 > T1 - Muscle relaxation: T2 > T1 - Aggression: T2 < T1 At home: - Sleep: T2 > T1 - Disturbance of siblings at bedtime: T2 < T1 - Rule-following re. bedtime activities: T2 > T1 - Rule-following re. homework completion: T2 > T1 - Positive behaviour with animals: T2 > T1 - Aggression with siblings and peers: T2 = T1 Retention—not reported	Poor
Hamama et al., 2011 [35]	Does AAT: (a) improve psychological distress, and (b) improve self-confidence and wellbeing, among teenage girls who were exposed to traumatic events?	N = 18 (100% female); 14–16 years. Ethnicity not reported. History of physical or sexual abuse (3–4 years before)—intervention group only. Control group had no history of abuse.	No information on dog(s) or handler(s) provided.	Pre- vs. post-test with non-concurrent control	Group intervention with AAT: 12 × 3 h sessions incorporating dog-focused and psychosocial intervention activities. Dog-focused activities included building trust, training, talking to and walking dog. n = 9. Comparison group: Classroom as usual (social games, learning control skills and public speaking). n = 9.	Wellbeing: self-report Coping with stressful life events: self-report PTSD Checklist—Civilian version (PCL-C): self-report. Measures diagnostic criteria for PTSD. Short Center for Epidemiologic Studies Depression Scale (SCESD): self-report. Measures depressive symptoms. Measures 1–4 administered at Time 1 (baseline) and Time 2 (post-intervention).	AAT (Measures 1–4: baseline (T1) vs. post-intervention (T2)). Wellbeing: T2 = T1 (d = 0.31) Coping with stressful life events: T2 = T1 (d = 0.19) PCL-C: T2 < T1 (d = 1.12) SCESD: T2 = T1 (d = 0.76). Retention—not reported Classroom as usual (Measures 1–4: baseline (T1) vs. post-intervention (T2)). Wellbeing: T2 = T1 (d = 0.74) Coping with stressful life events: T2 = T1 (d = 0.35) PCL-C: T2 = T1 (d = 0.08) SCESD: T2 = T1 (d = 0.05). Retention—not reported AAT vs. Classroom as usual (Measures 1–4: difference in mean scores at post-intervention (T2)). Wellbeing: AAT = Classroom as usual (d = 0.83) Coping with stressful life events: AAT = Classroom as usual (d = 0.36). PCL-C: AAT = Classroom as usual (d = 0.42) SCESD: AAT = Classroom as usual (d = 0.52). Retention—not reported	Poor
Muela et al., 2019 [40]	Is AAT effective in treating behavioural and emotional problems in children who have been exposed to domestic violence?	N = 19 (32% female); 6–15 years. Ethnicity not reported (although, all from Navarre region in north-east Spain). Exposed to domestic violence perpetrated by father or mother’s partner.	N = 4 (3× Labradors; 1 Golden Retriever) trained as therapy dogs. No information on handler(s) provided.	Pre- vs. post-test	‘Leaving a Mark’ AAT program: 14 × 1 h individual sessions. Program designed using principles of TF-CBT and AAT for childhood trauma. Modules, into which dog and dog-related activities are integrated, focus on establishing a secure base, psychoeducation, arousal regulation and stress management, and emotion expression and regulation.	1. Child Behaviour Checklist (CBCL): caregiver report. Measures behavioural and emotional problems across two broad syndrome subscales: (a) Internalising problems (b) Externalising problems. And two additional subscales: (c) Post-traumatic stress problems (d) Affective and behavioural dysregulation Measure administered at Time 1 (baseline) and Time 2 (post-intervention).	Leaving a Mark (Measures 1a–1d: baseline (T1) vs. post-intervention (T2)). 1a. Internalising problems: T2 < T1 (d = 0.38). 1b. Externalising problems: T2 = T1 (d = 0.19). 1c. Post-traumatic stress problems: T2 < T1 (d = 0.43) 1d. Affective and behavioural dysregulation: T2 = T1 (d = 0.27). Retention—not reported	Fair
Signal et al., 2017 [23]	Is an AAT program effective in reducing PTSD symptomatology in children who have experienced sexual abuse?	N = 20 (40% female); 5–12 years. 65% Caucasian, 35% Indigenous Australian. Experienced sexual abuse.	N = 1 trained therapy dog. No information on breed provided. Children interacted with dog in presence of dog’s handler/owner.	Multiple-baseline pre- vs. post-test	AAT: 10× sessions—Initial 3 sessions children attended RSPCA shelter and interacted in pairs with therapy dog in presence of handler for approx. 20–30 min—each of these 3 sessions had specific therapeutic objectives and activities designed and delivered by shelter education officer and program staff. Remaining 7 group sessions with assigned program social workers to transfer skills from animal to human interactions.	1. Trauma Symptom Checklist for Young Children (TSCYC): caregiver report. Measures PTSD-related symptoms. Alongside calculation of: (a) PTS Total score, specific subscales examined for this study included: (b) Intrusion (c) Aversion (d) Arousal (e) Dissociation. Measure administered at Times 1 (intake), 2 (program commencement) and 3 (post-intervention).	AAT (Measures 1a–1e: intake (T1) vs. program commencement (T2) vs. post-intervention (T3)). 1a. PTS Total score: T1 = T2 (d = 0.12). T3 < T2 (d = 0.72). 1b. Intrusion: T1 = T2 (d = 0.15). T3 < T2 (d = 0.60). 1c. Aversion: T1 = T2 (d = 0.08). T3 < T2 (d = 0.68). 1d. Arousal: T1 = T2 (d = 0.10). T3 < T2 (d = 0.73). 1e. Dissociation: T1 = T2 (d = 0.14). T3 < T2 (d = 0.50). Retention—not reported	Good

### 3.1. Is the Research of Sufficient Quality, and Is There Consistency in and Comprehensiveness of Descriptions of Dog-Assisted Therapy Program Theory and Components?

Two of the seven included studies received a quality rating of ‘good’. Just one of these utilised a randomised control design [36], while the other was a pre-post intervention design with no control [23]. Three of the remaining studies received a quality rating of ‘fair’, and two studies received a ‘poor’ rating, primarily due to methodological and reporting shortcomings, including noncomparative controls, limited descriptions of therapeutic activities and measurement methods, and the absence of statistical power calculations.

Most studies did not provide complete information either on the dog and its level of training or on the dog’s handler(s), including whether the handler was the therapist or a third party and how the presence of a third party was managed in the clinical setting. The two exceptions were Allen et al. [36], who worked with five Labrador retrievers trained as assistance dogs whose handlers observed the animals in sessions from behind a one-way mirror, and Eggiman [39], who was both handler and therapist and employed a poodle that had been trained as a therapy dog. No studies (excluding Eggiman et al., 2006 [39] where the therapist was a trained therapy dog handler) provided information on whether or how the therapist(s) was trained in the principles or methods of dog-assisted therapy.

Studies also varied in the degree to which they provided information on how the dogs were integrated into the therapeutic treatment. Three studies (c. 43%) reported dogs being employed as an adjunct to a primary therapeutic approach [36,38,39]. Allen et al. [36], described the dogs as being included as an adjunct to individual TF-CBT, but aside from child-led interactions, the clinician offered interaction with the dog only as a coping skill and did not otherwise include the dog in any of the session’s activities. Similarly, Eggiman [39] described the therapy dog as being integrated into individual CBT; however, only the first session was described in detail, with an explanation of the rules, getting to know the dog, petting and talking to the dog, and telling a therapeutic story about the dog’s behaviour, prior to closing the session and saying goodbye to the dog. Beyond this first session, details of the methods by which the dog was integrated into therapeutic activities, if at all, beyond simple presence in the sessions, were not provided. Dietz et al. [38], meanwhile, included dogs in conjunction with a pre-existing 12-week group therapy program, and compared two treatment approaches. The first approach involved therapy dogs and their handlers visiting the group treatment location once per month across four separate visits, where they were present in the lobby for 30 min prior to the group therapy session and then joined the group for 10–15 min for an ‘introductory activity’. The second approach followed this same format, but stories and questions relating to the dog and the wider session purpose were discussed in the group sessions following the therapy dog visits.

The remaining studies described interventions that were specifically designed as AAT programs, and which were conducted as either a stand-alone treatment program [23,40] or in combination with other forms of treatment [35,37]. Muela et al. [40] provide a comprehensive description of the Leaving a Mark program, which was developed using the principles of TF-CBT and animal-assisted intervention for childhood trauma, comprising five modules (i.e., establishing a secure base, psychoeducation, arousal regulation and stress management, emotion expression and regulation, and conclusion) delivered individually across 14 weekly, one-hour sessions. Activities that involve the therapy dog are described across each module, including exercises focusing on arousal regulation and stress management and an emotion identification activity that involves the dog fetching different coloured balls that were assigned to represent different emotions. Another stand-alone program is described by Signal et al. [23], whereby children were initially involved in three 90 min small-group sessions at a local Royal Society for the Prevention of Cruelty to Animals shelter with a therapy dog and handler, followed by seven sessions of group work with social workers that sought to transfer skills and concepts from animals to human interactions. Although Signal et al. [23] indicate that the three initial therapy dog sessions had specific therapeutic objectives and activities, no further information is provided on the theoretical basis or treatment principles underpinning the 10-week program.

Balluerka et al. [37] describe a 12-week treatment program that was developed based on psychotherapeutic models including psychotherapy for childhood trauma and attachment-based psychotherapy, and which is delivered across 34 sessions during 2-day overnight stays, once per week, at a farm in northern Spain. The treatment involved a mixture of individual and group sessions across six treatment blocks, including establishing a secure base, identifying, understanding, and verbalising emotions, emotional regulation, interpersonal relationships, self-esteem and self-competence, and therapy completion. Five sessions of the initial treatment block involved the introduction of the therapy dog, and some free, unstructured interaction with the dog. Subsequent therapy blocks involved other therapy animals, primarily horses, and aside from ‘living’ with participants during their weekly overnight stays, the therapy dog was not described as being involved in further therapeutic processes. Participants also continued to receive individual psychotherapy before, during, and after the animal-assisted program, but this was not further described.

Finally, Hamama et al. [35] described a 12-week program guided by a social worker and animal-assisted therapist, involving weekly 3 h group sessions that each revolved around a specific group intervention topic and associated activities with therapy dogs. Intervention topics included group introductions, trust-building, control skills and empowerment, sharing of hard feelings, and treatment closure. Activities involving the therapy dogs included trust-building exercises with a specific dog, training the dog to respond to verbal commands, and talking to the dog about feelings during a walk. There is no description of the theoretical basis or therapeutic model underpinning the program.

### 3.2. Is Dog-Assisted Therapy an Effective Treatment for Children Presenting with Complex Trauma Exposure, and Does It Have Any Beneficial Impact over and above Standard Therapeutic Treatments?

Studies assessed a range of complex trauma symptomology, including internalising (i.e., emotional) concerns, externalising (i.e., conduct) concerns, post-traumatic stress/PTSD, depression, anxiety, maladaptive coping skills, and personal and school-level maladjustment. Each are considered below.

#### 3.2.1. Internalising Concerns

Three quantitative studies examined internalising concerns. All three studies showed improvements among participants of dog-assisted therapy on internalising symptoms, with small to large effects, when compared to baseline [36,38,40]. In Dietz et al. [38], improvements in participants’ anger were shown only among those in the “dogs with stories” intervention group and not in the “dogs with no stories” intervention group.

Two of the three studies that examined internalising concerns compared dog-assisted therapy to a control condition. Dietz et al. [38] found improvements in terms of small to medium effects for anger concerns among the dog-assisted therapy groups over and above the control group, while Allen et al. [36] found no difference in improvements in emotional problems between the dog-assisted therapy and control groups.

#### 3.2.2. Externalising Concerns

Three quantitative studies and one qualitative study examined externalising concerns. One of the three quantitative studies showed improvements among participants involving dog-assisted therapy on externalising problems, with a small effect, when compared to baseline [36]. Two quantitative studies showed no change in externalising symptoms for dog-assisted therapy from baseline to post-intervention [37,40].

Two of the three quantitative studies compared dog-assisted therapy to a control condition, and neither showed differences in change for externalising symptom scales between the intervention and control groups [36,37]. However, one of these studies did find that children’s hyperactivity, a subscale of the externalising symptom scale, improved at a greater rate among dog-assisted therapy participants than among the control group [37].

One qualitative study also examined externalising concerns, with changes observed in conduct problems following the client’s exposure to dog-assisted therapy improved rule-following and decreased behavioural disturbances, particularly associated with bedtime routines, and increased positive behaviour with animals [39]. There were no observed changes in the client’s aggressive behaviour with siblings or peers [39].

#### 3.2.3. Post-Traumatic Stress

Five quantitative studies utilised measures of children’s post-traumatic stress. All five studies reported improvements in post-traumatic stress among dog-assisted therapy groups from baseline to post-intervention, with small to large effects [23,35,36,38,40]. Dietz and colleagues [38] reported a small effect for post-traumatic stress symptom changes among their ‘dogs + no stories’ intervention group, and a large effect for post-traumatic stress changes among their ‘dogs + stories’ intervention group. Allen et al. [36] found a large effect for change in caregiver-reported post-traumatic stress among the dog-assisted therapy group but no change in self-reported post-traumatic stress.

Three of the five studies that examined post-traumatic stress compared dog-assisted therapy to a control group. One of these studies found a greater improvement in post-traumatic stress symptoms among intervention participants than among the control group. This study found that participants in a ‘dogs + stories’ group showed greater change in post-traumatic stress, with a medium effect, than participants in a ‘dogs + no stories’ group. This group, in turn, showed greater change in post-traumatic stress, with a small effect, than a control group [38]. Allen et al. [36] found no difference in improvements in caregiver-reported post-traumatic stress between intervention and control groups, and a greater decrease in self-reported post-traumatic stress among the control group than the intervention group. The third study showed no difference in post-traumatic stress at Time 2 between the intervention group and a control group with no recorded history of trauma exposure.

#### 3.2.4. Depression

Three quantitative studies examined depressive symptoms. One of these found an improvement in depression among intervention group participants when compared with a baseline [38]. In this study, a ‘dogs + no stories’ intervention group showed a medium effect on depressive symptoms, and a ‘dogs + stories’ intervention group showed a large effect [38]. Two quantitative studies showed no change in depressive symptoms for dog-assisted therapy from baseline to post-intervention [35,36].

All three studies included comparisons of dog-assisted therapy with a control group. One of these studies found that intervention group participants improved in terms of their depressive symptoms to a greater degree than control participants [38]. The other two studies showed no difference between intervention and control groups in measures of depression.

#### 3.2.5. Anxiety

Two quantitative studies and one qualitative study included measures of anxiety. Of the two quantitative studies, one showed an improvement in anxiety among intervention group participants when compared with baseline [38]. In this study, a ‘dogs + no stories’ intervention group showed a small effect on anxiety, and a ‘dogs + stories’ intervention group showed a large effect [38]. The other quantitative study found no change in anxiety among intervention participants [36].

The two quantitative studies that examined anxiety also compared the dog-assisted therapy groups with a control. Dietz et al. [38] found that participants in the ‘dogs + stories’ group improved in terms of anxiety to a greater extent than participants in the ‘dogs + no stories’ group, with a medium effect, and that the ‘dogs + no stories’ group improved in terms of anxiety to a greater extent than participants in the control group, with a small effect.

In Eggiman’s [39] case study, reports were made of the client’s increased relaxation during therapy with the dog present, as well as reduced anxiety behaviours surrounding bedtime routines following participation in dog-assisted therapy.

#### 3.2.6. Maladjustment and Maladaptive Coping Skills

Two quantitative studies and one qualitative study reported on client adjustment and coping skills. One of the two quantitative studies showed improvements in adjustment and coping skills among intervention participants when compared to baseline. In this study, Balluerka et al. [37] found improvements in dog-assisted therapy participants for teacher-reported adaptive skills, with a large effect, and residential carer-reported adaptive skills, with a medium effect. There were no changes found from baseline to post-intervention for self-reported clinical maladjustment or personal adjustment, or teacher or self-reported school maladjustment [37]. The other quantitative study found no change among intervention participants in terms of self-reported wellbeing or coping when compared to baseline [35]. Additionally, when compared with the control group, Balluerka and colleagues [37] found a greater positive change for teacher-reported adaptive skills among their intervention group but not for residential carer-reported adaptive skills.

In Eggiman’s [39] case report, the client exposed to dog-assisted therapy was observed as showing increased attentive and focused behaviours in sessions.

### 3.3. Can Dog-Assisted Therapy Decrease Attrition and Increase Therapy Completion Rates?

Only one of the seven included studies reported the assessment of therapy retention rates as a research aim. Allen et al. [36] reported that three participants in their TF-CBT control group failed to complete treatment (21%), compared to just one participant (7%) in the TF-CBT + AAT group, which was a nonsignificant difference. Signal et al. [23] did not formally assess therapy completion rates but noted the adherence rate to their AAT program as being close to 90%, with most participants completing the treatment program. Signal et al. [23] did not include a program control group with which to directly compare adherence or completion outcomes. Balluerka et al. [37] noted that four participants did not complete treatment in their AAP program, with two of the enrolled participants being moved to special treatment care sessions, one being expelled for not following rules, and the fourth choosing not to continue treatment. There was no reported dropout from their psychotherapy control group [37].

## 4. Discussion

The aims of this review were to examine the impact of dog-assisted therapy on the treatment of complex trauma in childhood, as well as its effect on treatment retention and completion, in the context of an assessment of program description, research quality and comprehensiveness. Only seven studies were found to meet the inclusion criteria for this review, showing the dearth of evidence that exists specifically relating to dog-assisted therapy and complex trauma treatment. Additionally, only one included study formally examined the impact of dog-assisted therapy on treatment retention. Studies varied in the ways in which dog-assisted therapy was incorporated into treatment, and many failed to report a sufficient level of detail about the dogs, their training, the handlers, and the activities in which dogs were integrated into the treatment. Studies did show that dog-assisted therapy may have some benefits for the treatment of childhood trauma, although results varied across studies, and methodological limitations of included studies indicate the need for further research to build certainty in the evidence base for dog-assisted therapy and complex trauma treatment.

Research has established that children who experience maltreatment show a range of behavioural, emotional, physiological, and cognitive symptoms. Previous reviews of AAT have shown positive impacts on complex trauma symptoms in both children and adults, including depressive, anxiety, and post-traumatic stress-related symptomology (e.g., [27,41]); however, to date, no reviews had specifically examined dog-assisted therapy in the treatment of complex trauma in childhood. Of the seven studies included in this review, most assessed internalising (i.e., emotional) and externalising (i.e., conduct) symptoms of complex trauma, along with post-traumatic stress. Overall, positive results were shown across all five studies that examined post-traumatic stress, with one inconsistent finding. Allen et al. [36] reported that caregiver-reported post-traumatic stress symptoms decreased following both TF-CBT and TF-CBT + AAT, but that self-reported post-traumatic stress decreased only among the TF-CBT control group. While our search was robust because we relied on PRISMA guidelines, it is possible that we may have found different publications if we had used different databases for the search terms. We think this is unlikely, however, because we identified studies both by database search and other methods (e.g., reviewing reference lists for included papers).

Two of the studies that examined internalising symptoms showed positive results [36,40], but the one study that included a control group found a similar positive impact among the comparison participants [36]. Dietz et al. [38] also reported a positive program impact on participants’ anger. Findings regarding externalising problems were inconsistent—Allen et al. [36] found only a small program impact on conduct concerns, while Muela et al. [40] found no impact on externalising concerns or behavioural dysregulation. Balluerka et al. [37] showed no overall impact on behavioural concerns but a positive result for teacher-reported hyperactivity.

Similarly mixed findings were reported regarding depressive and anxiety symptoms—while one study showed a positive impact of dog-assisted therapy on both depression and anxiety [38], two further studies found no change in depressive symptoms [35,36] and Allen et al. [36] reported reduced anxiety only among those in the TF-CBT control group. There were mostly no reported changes in studies that examined maladjustment and maladaptive coping measures, although there were beneficial results found for teacher-reported adaptive skills in Belluerka et al.’s [37] animal-assisted psychotherapy program. In their qualitative case study, Eggiman [39] reported client improvement across a range of behaviours, including increased attention and focused behaviour in sessions, but Eggiman [39] did indicate that the client’s aggressive behaviour with siblings and peers outside of sessions did not change following engagement in dog-assisted therapy.

There are several possible reasons for the variability in results found across included studies—one being the range of measures used to assess trauma symptomology. For example, while Dietz et al. [38] found a positive impact of dog-assisted therapy on depressive symptoms using the depression scale of the Trauma Symptom Checklist, inconsistent findings were shown across studies that utilised the Mood and Feelings Questionnaire [36] and the Short Center for Epidemiologic Studies Depression Scale [35]. The range in terms of the quality of the included studies is also a potential explanation for the contradictory findings. For example, of the three studies that reported a change in participant maladjustment and maladaptive coping skills, two were rated as having ‘poor’ levels of quality while one was rated as ‘fair’. The low level of quality and associated certainty from several of the included studies likely reflect challenges in conducting research in this area. As noted by Serpell et al. [42], in AAT research, it is impossible to blind participants to their group, and the client’s previous experiences with, and perceptions of, animals may influence AAT outcomes. Furthermore, the public’s interest in ‘good news stories’ about animals helping humans can put pressure on researchers to only publish positive findings [42]. Nonetheless, these findings may not be sufficiently indicative of how dog-assisted therapy may impact client adjustment or adaptive coping behaviours.

It is also likely that the role of the dog in the different therapy programs may explain the inconsistencies in the reported results. Across studies, the role of the dog in what was commonly termed ‘dog-assisted therapy’ was shown to fit along a spectrum from passive integration (i.e., the dog is present in the room) to active integration (i.e., the role of the dog is planned, and the dog is included in therapeutic activities). It is reasonable to assume that active engagement may facilitate the effects of dog-assisted therapy, but in many cases, the role of the dog was not clearly explained at all. To fully understand the impact of dog-assisted therapy, the level of involvement of the dog in therapeutic activities must be considered and reported in research studies as a potential moderating factor.

Alongside the lack of therapeutic integration information, another pervasive issue is the lack of detail that is included across studies on the dog(s), their level of training, their handler(s), and the handlers’ or therapists’ training. Of the seven included studies, four provided details on the breed and training of the dogs used, but only three provided details on the dog’s handler/therapist and any training that they may have received in dog-assisted therapy techniques. In the case where external handlers attended with therapy dogs, no information was provided in any study as to whether the therapists received training in integrating the dog into their clinical practise (e.g., [36]). The different models of therapy used, including engagement of external handlers who brought therapy dogs into a treatment program (e.g., [38]) as compared with a therapist who was presumably trained alongside their own therapy dog to integrate the dog into their existing treatment model (e.g., [39]), limits our ability to draw conclusions regarding effective parameters of dog-assisted therapy. Future research should explore this factor as a means of better understanding how dogs may best be employed in dog-assisted therapy for complex trauma.

Various theoretical discussions of the ways in which dogs may facilitate social relationships and empathy lend themselves to the hypothesis that the bond developed between a child trauma sufferer and a therapy dog may increase engagement in therapy, thereby increasing treatment retention and completion rates. Despite this, however, only one of the studies included in this review directly assessed program impact on therapeutic treatment completion, finding a nonsignificant impact of dog-assisted therapy [36]. While not directly assessing retention, Signal et al. [23] reported strong program adherence in their dog-assisted therapy program, which they discussed as being nontypical for studies involving trauma treatment. Future research examining the impact of dog-assisted therapy should prioritise examination of therapeutic completion, particularly as evidence-based treatments do exist for complex trauma, and dog-assisted therapy may be of particular benefit as a complement to these treatments by enabling increased adherence and retention. Dog-assisted therapy may be most effective in its facilitation of a safe, empathetic, secure, and warm environment [43]. Whilst such a broad explanation is consistent with various theoretical discussions, none of the studies included in this review allowed for a direct investigation of these theoretical explanations. Future research should make this a priority, for example, by measuring oxytocin, which has been proposed to be an important biological component facilitating dog-assisted therapy [22] and including measures indicative of social competency and the role of the dog in the development of therapeutic bond strength (i.e., forming relationships, trust, and secure attachment).

Additionally, while evidence-based treatments do exist for complex trauma treatment, few studies have made use of these existing treatment models in the development of dog-assisted therapy programming. One exception is Allen et al. [36], who studied the existing gold-standard treatment TF-CBT alone and with the integration of a dog into TF-CBT. Interestingly, Allen et al. [36] reported few additional benefits of AAT to TF-CBT alone and indicated that, in some cases, the presence of the dog may have negatively impacted client treatment through increased avoidance of therapeutic activities. The model of dog-assisted therapy used for this study did show some limitations, however, with external handlers making their dogs available to therapists who were not reported to have received any training in dog-assisted therapy and who did not actively integrate the dog into therapeutic activities aside from offering the dog as a comfort or coping mechanism during sessions. It is possible that with training on dog-assisted therapy techniques, these therapists may have been able to avoid any negative impact or distraction of the dog in their sessions.

Finally, no studies included in this review reported any long-term impacts following engagement in dog-assisted therapy, with each study making use of only immediate post-intervention measures. It will be important to explore the possible long-term effects of dog-assisted therapy as research in this area develops.

It is evident that dog-assisted therapy may be a viable complement to the treatment of children with complex trauma, but there is a lack of methodologically rigorous research in this area, as well as a lack of understanding as to what dog-assisted therapy should optimally consist of. This is important given that already established approaches, including TF-CBT, are associated with high dropout rates, which essentially nullifies the effectiveness of such approaches for those unable to continue with them (e.g., [44]). It is important that future research continues to examine the impact of dog-assisted therapy for the treatment of complex trauma, not just in terms of its impact on trauma symptomology but also on the components of dog-assisted therapy that most directly impact child outcomes, as well as the factors that may mediate or moderate outcomes, including treatment retention and completion.

## Figures and Tables

**Table 1:** List of terms to identify dog-assisted interventions for childhood trauma in database searches. An asterisk * is used in databases to locate any word with that stem (e.g., ‘assist *’ may show terms like ‘assisted’, ‘assisting’, ‘assistance’, and ‘assist’).

Animal assist *	Child * sex * abuse	Maltreatment
AAT	CSA	Child * abuse
Canine assist *	Child trauma	Neglect
CAT	Trauma	Adverse child * experiences
Dog assist *	Abuse	Maltreat *
Support dog		

## Data Availability

For the results of the literature review, please contact D.B. The data are not publicly available due to ethics requirements.
